# *Myroxylon pereirae* (balsam of Peru): Still worth testing?

**DOI:** 10.1111/cod.13839

**Published:** 2021-05-03

**Authors:** Fabrizio Guarneri, Monica Corazza, Luca Stingeni, Cataldo Patruno, Maddalena Napolitano, Paolo D. M. Pigatto, Rosella Gallo, Antonio Cristaudo, Paolo Romita, Annamaria Offidani, Donatella Schena, Nicola Milanesi, Giuseppe Micali, Myriam Zucca, Caterina Foti, SIDAPA Study Group, Alessandro Borghi, Katharina Hansel, Giovanni Damiani, Ilaria Trave, Emanuela Martina, Giacomo Dal Bello, Massimo Gola, Maria L. Musumeci, Viviana Piras

**Affiliations:** ^1^ Department of Clinical and Experimental Medicine University of Messina Messina Italy; ^2^ Sezione di Dermatologia, Dipartimento di Scienze Mediche Università degli Studi di Ferrara Ferrara Italy; ^3^ Dermatology Section, Department of Medicine University of Perugia Perugia Italy; ^4^ Department of Health Sciences University Magna Graecia of Catanzaro Catanzaro Italy; ^5^ Department of Health Sciences "V. Tiberio" University of Molise Campobasso Italy; ^6^ IRCCS Istituto Ortopedico Galeazzi, Department of Biomedical, Surgical and Dental Sciences University of Milan Milan Italy; ^7^ Section of Dermatology ‐ Department of Health Sciences University of Genoa, Ospedale Policlinico San Martino – IRCCS Genoa Italy; ^8^ UOSD Dermatologia MST, Ambientale, Tropicale e Immigrazione, Istituto Dermatologico San Gallicano (IRCCS) Rome Italy; ^9^ Section of Dermatology, Department of Biomedical Science and Human Oncology University of Bari Bari Italy; ^10^ Clinica Dermatologica, Dipartimento di Scienze Cliniche e Molecolari Università Politecnica delle Marche Ancona Italy; ^11^ Section of Dermatology and Venereology, Department of Medicine University of Verona Verona Italy; ^12^ Allergological and Occupational Dermatology Unit, Department of Health Sciences AUTC and University of Florence Florence Italy; ^13^ Dermatology Clinic University of Catania, PO G. Rodolico, AOU Policlinico‐Vittorio Emanuele Catania Italy; ^14^ Dermatologic clinic, University Hospital S. Giovanni di Dio Cagliari Italy

**Keywords:** balsam of Peru, fragrance allergy, fragrance mix 1, *Myroxylon pereirae*, patch test

## Abstract

**Background:**

Because *Myroxylon pereirae* (MP), or balsam of Peru, is nowadays almost not used “as such,” and fragrance mix 1 (FM1) apparently is more sensitive in detecting fragrance allergy, the usefulness of testing MP in baseline series was recently questioned.

**Objectives:**

Identification of the number of clinically relevant patch test reactions to MP not detected by FM1.

**Methods:**

Retrospective analysis of 12 030 patients patch tested with MP and FM1 for contact dermatitis between January 2018 and December 2019 in 13 Italian dermatology clinics.

**Results:**

Four hundred thirty‐nine patients (3.6%) had a positive patch test reaction to MP; 437 (3.6%) had a positive patch test reaction to FM1. Positive reactions to both MP and FM1 were observed in 119 subjects (1.0%), 310 (2.6%) reacted to MP only, 304 (2.5%) to FM1 only, 5 to MP and sorbitan sesquioleate (SSO), 9 to FM1 and SSO, and 5 to MP, FM1, and SSO. Single sensitizations were clinically relevant in 75.2% of cases for MP (62.9% current, 12.3% past) and 76.3% for FM1 (70.1% current, 6.2% past).

**Conclusions:**

Based on our results, MP appears to be still worth testing along with FM1 in baseline series, because it allows detection of a remarkable number of fragrance allergies, often relevant, which would be otherwise missed.

## 1 INTRODUCTION

*Myroxylon pereirae* (MP), also known as balsam of Peru (CAS no. 8007‐00‐9), is an aromatic, fixative and mild antiseptic, antifungal, and antiparasitic resin, obtained from the bark of *Myroxylon balsamum var. pereirae*.^1^, ^2^ MP has been a frequent contact sensitizer in the past, and for this reason its use “as such” has been almost abandoned; however, 4%–8% of patients tested with baseline series still show positive reactions to MP.^1^ Although with a certain variability in its composition, MP contains several fragrance chemicals^1^ and is considered a marker of fragrance allergy. Positive patch tests to MP and fragrance mix 1 (FM1) are frequently associated because of shared components. This fact, and the higher sensitivity of FM1 in detecting fragrance allergy, led some authors to propose elimination of MP from baseline series.^1^ In a recent article, de Groot^1^ suggested a path to achieve a definitive conclusion on this matter, composed of four sequential studies. In his words, “Study 1 determines the percentage of single positive reactions to MP (ie, with negative results for FM I) and how many of these are relevant, either by indicating fragrance allergy or from contact with products actually containing MP. These data can give an indication of the added value of MP and whether MP qualifies for continued inclusion in the European baseline series.”^1^ Following these suggestions, we performed a nationwide study aimed to define the “added value” of testing MP along with FM1, that is, the number of clinically relevant positive reactions to MP not detected by FM1, which represent cases of fragrance allergy that would be otherwise missed.

## 2 METHODS

We retrospectively examined the data of 12 030 consecutive patients (4110 male and 7920 female, mean age 47.3 ± 17.2 years, range 13–80) who were patch tested for contact dermatitis between January 2018 and December 2019 in 13 dermatology clinics homogeneously distributed across Italy. All patients had underwent patch testing with the SIDAPA (Società Italiana di Dermatologia Allergologica Professionale e Ambientale) baseline series,^3^ in Haye's Test Chambers (Haye's Service, Alphen aan den Rijn, The Netherlands) on SOFFIX tape (Artsana, Grandate, Italy). Readings were performed by experienced dermatologists in line with national and international guidelines,^3^ on day 2 (D2), D4, and D7, with patients asked to return if late reactions occurred. When appropriate on the basis of clinical history and results, further patch tests with specific haptens and/or suspected products were performed subsequently, with an interval of at least 1 month. Cases of positive reactions to MP and/or FM1, associated or not with allergy to sorbitan sesquioleate (SSO), were selected (irritant or doubtful reactions were not considered). The assessment of clinical relevance of positive MP patch test was based on fulfilling the following three criteria: patient's history of possible exposure to scented products, clinical examination, and remission of dermatitis after the patient stopped using scented products.^3^ For the purpose of this study, only clearly positive reactions (ie, +, ++ or +++) were considered. Equally tests with ?+ reactions were repeated and readings were performed up to D7. Microsoft Excel (Microsoft, Redmond, Washington) was used for statistical analysis (Student *t*‐test for continuous data, chi‐square test for categorical data). *P* < .05 with Bonferroni correction was considered significant.

## 3 RESULTS

**FIGURE 1:**
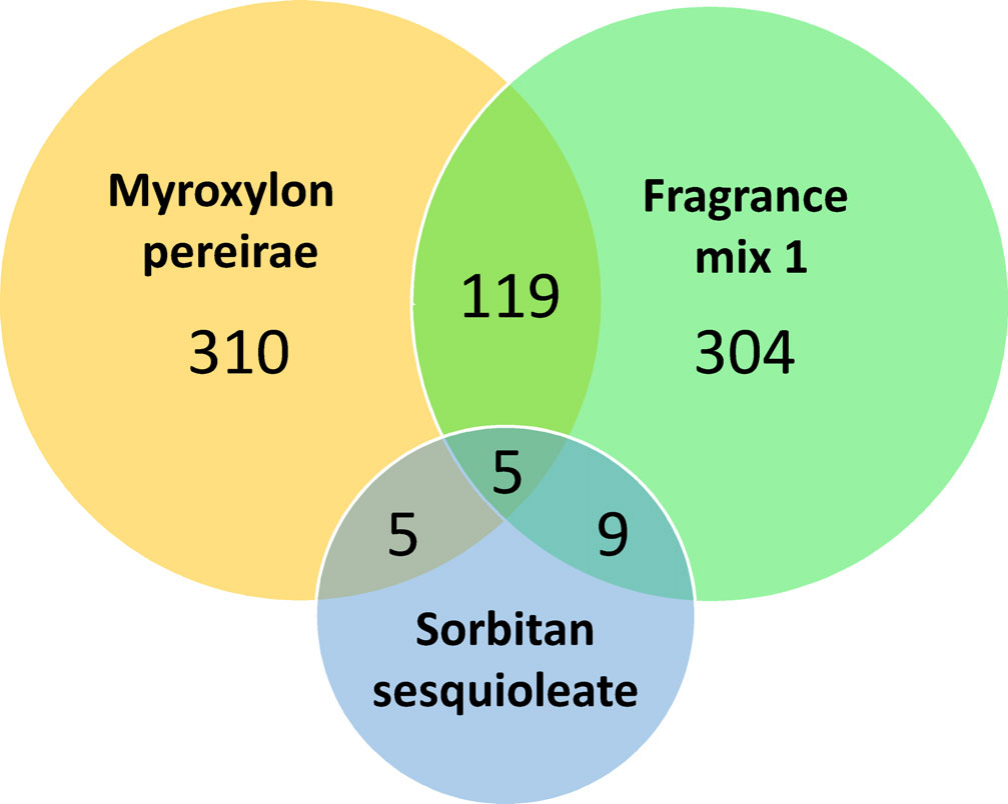
Number of patients with patch tests that were positive to *Myroxylon pereirae* (balsam of Peru) and/or fragrance mix 1, associated or not with allergy to sorbitan sesquioleate. (Note: the circle representing sorbitan sesquioleate is not in scale.) Allergy to sorbitan sesquioleate is shown because this substance can be a component of both fragrance mix 1 and *Myroxylon pereirae* preparations used for patch tests

Among the 12 030 patients, 752 (6.3%) were included in the study; 439 (3.6%) had a positive patch test reaction to MP and 437 (3.6%) to FM1. Positive reactions to both MP and FM1 were observed in 119 subjects (1.0%), whereas 310 (2.6%) reacted to MP only and 304 (2.5%) to FM1 only; the remaining 19 were positive to SSO in addition to MP and/or FM1 (5 MP + SSO, 9 FM1 + SSO, 5 MP + FM1 + SSO) (Figure 1). Table 1 shows scoring and clinical relevance of positive patch test reactions to MP and/or FM1. Comparison between centers did not show significant differences in the relative frequency of scores attributed to patch test reactions. Single sensitizations were clinically relevant in 75.2% of cases for MP (62.9% current, 12.3% past) and 76.3% for FM1 (70.1% current, 6.2% past).

**TABLE 1:** Results and clinical relevance of patch tests in patients who were positive to *Myroxylon pereirae* (n = 310), fragrance mix 1 (n = 304), or both (n = 119)

Result of patch test	Number of positive reactions (%)	
**Myroxylon pereirae only (n = 310)**	**Relevant**	**Not relevant**
+	95 (30.6)	56 (18.1)
++	126 (40.6)	21 (6.8)
+++	12 (3.9)	—
**Fragrance mix 1 only (n = 304)**	**Relevant**	**Not relevant**
+	102 (33.6)	47 (15.5)
++	116 (38.2)	25 (8.2)
+++	14 (4.6)	—
***Myroxylon pereirae*/fragrance mix 1 (n = 119)**	**Both relevant**	**Both not relevant**
+ / +	12 (10.1)	4 (3.4)
+ / ++	10 (8.4)	1 (0.8)
+, not relevant / ++, relevant	2 (1.7)	
+ / +++	7 (5.9)	—
++ / +	12 (10.1)	1 (0.8)
++ / ++	45 (37.8)	2 (1.7)
++ / +++	10 (8.4)	—
+++ / +	3 (2.5)	—
+++ / ++	6 (5.0)	—
+++ / +++	4 (3.4)	—

The mean age of patients who were positive to MP only was 55.8 ± 16.3 years, higher than that of patients who were positive to FM1 only (48.4 ± 19.0 years; *P* < .001) or with co‐reactivity to MP and FM1 (51.6 ± 17.9 years; *P* = .027, not significant after Bonferroni correction). Female patients were more represented among patients who were positive to FM1 only (n = 219, 72.0%) than among those positive to MP only (n = 188, 60.7%; *P* = .0028) or to MP and FM1 (n = 72, 60.5%; *P* = .021, not significant after Bonferroni correction). A personal history of atopy (cutaneous and/or respiratory) was present in 96 patients who were positive to MP only (31.0%), 134 positive to FM1 only (44.1%; *P* < .001 vs the previous group), and 46 positive to both MP and FM1 (38.7%). No significant differences between groups were observed for frequency of facial, leg, or hand dermatitis.

## 4 DISCUSSION

Diagnosis of fragrance allergy is often challenging. The high (and increasing) number of substances possibly involved is an important cause of such difficulties. Based on scientific literature, the European Union defined a list of 26 fragrances identified as contact allergens in humans, the use of which must be explicitly declared in the label of any product marketed in member countries.^2^ Baseline series contain some markers for fragrance allergy: in the European and in the SIDAPA series, these are MP, FM1, FM2, and hydroxyisohexyl 3‐cyclohexene carboxaldehyde.^3^ MP is a mixture of multiple and yet not completely defined substances, which includes components of FM1 (among other fragrances of baseline series, only farnesol, a component of FM2, may be present in some MP extracts, but only in traces).^1^ This fact, together with the substantial elimination of MP “as such” from consumer products (only some extracts and distillates can be used, with restrictions, in products that may come in contact with skin), led some authors to question whether the use of MP in baseline series may detect cases of fragrance allergy that are not already revealed by FM1.^1^

Our data suggest that positive patch test reactions to MP and to FM1 frequently do not coincide, and many relevant allergies would be missed if MP was excluded from baseline patch test series: in our population, 2.6% were positive only to MP and among patients with clinically relevant allergy to MP (n = 342), 233 (68.1%) were negative to FM1. Our data also show that coreactivity to MP and FM1 can be explained by sensitization to SSO only in few cases, as already suggested in the literature.^1^, ^4^

Crude MP cannot be used in perfumes and cosmetics, whereas some extracts and distillates are allowed with restrictions^1^: this might explain the higher percentage of women, main consumers of such products,^5^, ^6^ among patients allergic to FM1 only.

Although the lack of coreactivity to FM1 in many patients allergic to MP is known, few studies tried to define the “added value” of testing MP in addition to FM1.^7^, ^8^, ^9^, ^10^ In three papers published in 1996,^7^ 1997,^8^ and 2001,^9^ Johansen et al. reported two cases of allergy to MP not associated with allergy to FM1 (relevance not explicitly stated), but only data concerning patients sensitized to perfumes (23, 37, and 33, respectively, of 335, 500, and 480 patch tested) were described in the papers. Cuesta et al.^10^ found, among 1253 routinely tested patients, 51 positive to MP but not FM1, 27 to FM1 but not MP, and 29 to both; however, the individual relevance of these reactions was not reported. In a subgroup of 54 patients tested also with a specific fragrance series, 3 of 12 single reactions to MP, 16 of 19 single reactions to FM1, and 11 of 17 co‐reactions to MP and FM1 were associated with reactions to other fragrances. These data differ remarkably from ours, but were obtained in a much smaller population. More recently, Uter et al. published a retrospective analysis of 40 709 patch tests performed in Information Network of Departments of Dermatology centers in a 4‐year period.^11^ Of these patch tests, 35 361 included MP, FM1, and FM2, and the frequencies of positive reactions to these three haptens were 7.82%, 7.17%, and 4.82%, respectively. As in our population, single reactions to MP (5.54%) or FM1 (4.89%) were much more frequent than simultaneous reactions to MP and FM1 (2.28%).^11^ Also in this case, however, the relevance of reactions to MP was not declared.

As reported by de Groot,^1^ use of MP in pharmaceutical, cosmetic, or consumer products is nowadays very limited, with no other known relevant sources of contact (the role of systemic intake through diet or other sources is debated), but MP is still a marker of fragrance allergy. A possible explanation, suggested by some authors,^1^, ^12^ is that MP might reveal the sensitization to fragrances (contained in cosmetics, perfumes, and so on) that are not “picked up” by FM1. However, the substances responsible for single allergic reactions to MP are currently largely unknown.^1^

A limitation of our study, shared with all others available in the literature to date, is its retrospective nature. Second, we considered only patients with patch tests that were positive to MP and/or FM1, without evaluating cases of possible co‐reactivity between MP and other markers of fragrance allergy included in the baseline series (FM2 and hydroxyisohexyl 3‐cyclohexene carboxaldehyde). However, the possibility that these substances may reveal a sensitization to components of MP not detected by FM1 appears remote: as mentioned above, at the present state of knowledge, farnesol (contained in FM2) is the only one of these “non‐FM1” fragrances of baseline series that may be contained in MP, although in traces and inconstantly.^1^ Thus the role of FM2 in the definition of the “added value” of testing MP in baseline series is expected to be rather limited. Consistently with this theoretical basis, Uter et al. reported that only 0.4% of 35 361 patients tested were simultaneously positive to MP and FM2 and negative to FM1.^11^

We chose to repeat patch tests with MP in all the patients with doubtful (?+) patch test reactions, performing readings up to D7, because these cases must be considered worth of attention in clinical practice, as they are sometimes true reactions, clinically relevant for disease.^13^ Similarly, to be more selective, we classified as not relevant all reactions for which a clear connection with present or past exposure could not be found; however, origin and clinical relevance of reactions to MP often remain unclear in daily practice, because patients are not always able to provide complete and reliable pertinent anamnestic data.

Future research should be focused on better definition of the components of MP: 30–40% of them are yet unknown resins and vegetal oils, potentially allergenic.^14^ Identification and separate testing of these components could reveal those with more frequent clinical relevance and not detected by FM1.

Another interesting line of clinical research could be to prospectively and systematically test the 26 highly allergenic fragrances, identified by the European Union,^2^ in all patients with suspected fragrance allergy. Indeed, some authors showed that, in a number of cases, patch tests with all fragrance screeners give negative results, and only tests with individual fragrances can reveal allergy (but, surprisingly, also vice versa).^15^, ^16^, ^17^, ^18^

## 5 CONCLUSIONS

Based on our results, we believe that at the current state of knowledge MP is still worth testing along with FM1 in baseline series, because it allows detection of a remarkable number of fragrance allergies, often relevant, which would be otherwise missed.

## CONFLICT OF INTEREST

The authors have no conflict of interest to declare.

## AUTHOR CONTRIBUTIONS

**Fabrizio Guarneri:** Conceptualization; data curation; formal analysis; project administration; resources; writing‐original draft; writing‐review & editing. **Monica Corazza:** Investigation; methodology; resources; validation; writing‐original draft. **Luca Stingeni:** Investigation; methodology; resources; validation; writing‐original draft. **Cataldo Patruno:** Investigation; methodology; resources; validation; writing‐original draft. **Maddalena Napolitano:** Investigation; methodology; resources; validation; writing‐original draft. **Paolo Pigatto:** Investigation; methodology; resources; validation; writing‐original draft. **Rosella Gallo:** Investigation; methodology; resources; validation; writing‐original draft. **Antonio Cristaudo:** Investigation; methodology; resources; validation; writing‐original draft. **Paolo Romita:** Investigation; methodology; resources; validation; writing‐original draft. **Annamaria Offidani:** Investigation; methodology; resources; validation; writing‐original draft. **Donatella Schena:** Investigation; methodology; resources; validation; writing‐original draft. **Nicola Milanesi:** Investigation; methodology; resources; validation; writing‐original draft. **Giuseppe Micali:** Investigation; methodology; resources; validation; writing‐original draft. **Myriam Zucca:** Investigation; methodology; resources; validation; writing‐original draft. **Foti Caterina:** Investigation; methodology; project administration; resources; supervision; validation; writing‐original draft; writing‐review & editing.

## Collaborators SIDAPA Study Group

Alessandro Borghi, Sezione di Dermatologia, Dipartimento di Scienze Mediche, Università degli Studi di Ferrara, Ferrara, Italy; Katharina Hansel, Dermatology Section, Department of Medicine, University of Perugia, Perugia, Italy; Giovanni Damiani, IRCCS Istituto Ortopedico Galeazzi, Department of Biomedical, Surgical and Dental Sciences, University of Milan, Milan, Italy; Ilaria Trave, Section of Dermatology ‐ Department of Health Sciences, University of Genoa, Ospedale Policlinico San Martino – IRCCS, Genoa, Italy; Emanuela Martina, Clinica Dermatologica, Dipartimento di Scienze Cliniche e Molecolari, Università Politecnica delle Marche, Ancona, Italy; Giacomo Dal Bello, Section of Dermatology and Venereology, Department of Medicine, University of Verona, Verona, Italy; Massimo Gola, Allergological and Occupational Dermatology Unit, Department of Health Sciences, AUTC and University of Florence, Florence, Italy; Maria L. Musumeci, Dermatology Clinic, University of Catania, PO G. Rodolico, AOU Policlinico‐Vittorio Emanuele, Catania, Italy; Viviana Piras, Department of Medical Sciences and Public Health, Unit of Dermatology, University of Cagliari, Cagliari, Italy.

## Data Availability

Data available on request due to privacy/ethical restrictions

## References

[1] de Groot AC. *Myroxylon pereirae* resin (balsam of Peru) ‐ a critical review of the literature and assessment of the significance of positive patch test reactions and the usefulness of restrictive diets. Contact Dermatitis. 2019;80(6):335‐353. 30843216 10.1111/cod.13263

[2] Directorate‐General for Internal Market, Industry, Entrepreneurship and SMEs (Small and Medium‐sized Enterprises) of the European Union . Cosmetic ingredient database (Cosing)—Ingredients and Fragrance inventory. http://data.europa.eu/euodp/data/dataset/cosmetic-ingredient-database-ingredients-and-fragrance-inventory. Accessed June 21, 2020.

[3] Stingeni L, Bianchi L, Hansel K, et al. Italian guidelines in patch testing ‐ adapted from the European Society of Contact Dermatitis (ESCD). G Ital Dermatol Venereol. 2019;154(3):227‐253. 30717577 10.23736/S0392-0488.19.06301-6

[4] Stingeni L, Tramontana M, Bianchi L, et al. Patch test with sorbitan sesquioleate in Italian consecutive patients: a 1‐year multicenter SIDAPA study. Contact Dermatitis. 2019;81(6):454‐456. 31328272 10.1111/cod.13359

[5] Lim S. The associations between personal care products use and urinary concentrations of phthalates, parabens, and triclosan in various age groups: the Korean National Environmental Health Survey Cycle 3 2015‐2017. Sci Total Environ. 2020;742:140640. https://pubmed.ncbi.nlm.nih.gov/32721747 32721747 10.1016/j.scitotenv.2020.140640

[6] Grand View Research . Perfume Market Size, Share & Trends Analysis Report by Product (Mass, Premium), by End User (Men, Women), by Distribution Channel (Offline, Online), by Region, and Segment Forecasts, 2019‐2025. San Francisco: Grand View Research; 2019.

[7] Johansen JD, Rastogi SC, Menné T. Contact allergy to popular perfumes: assessed by patch test, use test and chemical analysis. Br J Dermatol. 1996;135(3):419‐422. 8949436

[8] Johansen JD, Rastogi SC, Andersen KE, Menné T. Content and reactivity to product perfumes in fragrance mix positive and negative eczema patients. Contact Dermatitis. 1997;36(6):291‐296. 9237007 10.1111/j.1600-0536.1997.tb00003.x

[9] Johansen JD, Frosch PJ, Rastogi SC, Menné T. Testing with fine fragrances in eczema patients: results and test methods. Contact Dermatitis. 2001;44(5):304‐307. 11298698 10.1034/j.1600-0536.2001.440510.x

[10] Cuesta L, Silvestre JF, Toledo F, Lucas A, Perez‐Crespo M, Ballester I. Fragrance contact allergy: a 4‐year retrospective study. Contact Dermatitis. 2010;63(2):77‐84. 20573166 10.1111/j.1600-0536.2010.01739.x

[11] Uter W, Geier J, Frosch P, Schnuch A. Contact allergy to fragrances: current patch test results (2005‐2008) from the information network of departments of dermatology. Contact Dermatitis. 2010;63(5):254‐261. 20731693 10.1111/j.1600-0536.2010.01759.x

[12] Trattner A, David M. Patch testing with fine fragrances: comparison with fragrance mix, balsam of Peru and a fragrance series. Contact Dermatitis. 2003;49(6):287‐289. 15025700 10.1111/j.0105-1873.2003.0264.x

[13] Bennike NH, Palangi L, Christensson JB, et al. Allergic contact dermatitis caused by hydroperoxides of limonene and dose‐response relationship‐a repeated open application test (ROAT) study. Contact Dermatitis. 2019;80(4):208‐216. 30378136 10.1111/cod.13168

[14] Riesmeier M, Mattonai M, Wong SS, et al. Molecular profiling of Peru balsam reveals active ingredients responsible for its pharmaceutical properties. Nat Prod Res. 2020;1‐6. 10.1080/14786419.2020.1753056 32316792

[15] van Oosten EJ, Schuttelaar ML, Coenraads PJ. Clinical relevance of positive patch test reactions to the 26 EU‐labelled fragrances. Contact Dermatitis. 2009;61(4):217‐223. 19825093 10.1111/j.1600-0536.2009.01605.x

[16] Heisterberg MV, Menné T, Johansen JD. Contact allergy to the 26 specific fragrance ingredients to be declared on cosmetic products in accordance with the EU cosmetics directive. Contact Dermatitis. 2011;65(5):266‐275. 21943251 10.1111/j.1600-0536.2011.01962.x

[17] Mann J, McFadden JP, White JM, White IR, Banerjee P. Baseline series fragrance markers fail to predict contact allergy. Contact Dermatitis. 2014;70(5):276‐281. 24731084 10.1111/cod.12171

[18] Ung CY, White JML, White IR, Banerjee P, McFadden JP. Patch testing with the European baseline series fragrance markers: a 2016 update. Br J Dermatol. 2018;178(3):776‐780. 28960261 10.1111/bjd.15949

